# Functional conservation of *Pax6 *regulatory elements in humans and mice demonstrated with a novel transgenic reporter mouse

**DOI:** 10.1186/1471-213X-6-21

**Published:** 2006-05-04

**Authors:** David A Tyas, T Ian Simpson, Catherine B Carr, Dirk A Kleinjan, Veronica van Heyningen, John O Mason, David J Price

**Affiliations:** 1 Genes and Development Group, Centres for Integrative Physiology and Neuroscience Research, University of Edinburgh, Hugh Robson Building, George Square, Edinburgh EH8 9XD, UK; 2 MRC Human Genetics Unit, Western General Hospital, Edinburgh EH4 2XU, UK

## Abstract

**Background:**

The Pax6 transcription factor is expressed during development in the eyes and in specific CNS regions, where it is essential for normal cell proliferation and differentiation. Mice lacking one or both copies of the *Pax6 *gene model closely humans with loss-of-function mutations in the *PAX6 *locus. The sequence of the Pax6/PAX6 protein is identical in mice and humans and previous studies have shown *structural *conservation of the gene's regulatory regions.

**Results:**

We generated a transgenic mouse expressing green fluorescent protein (GFP) and neomycin resistance under the control of the entire complement of human *PAX6 *regulatory elements using a modified yeast artificial chromosome (YAC). Expression of GFP was studied in embryos from 9.5 days on and was confined to cells known to express Pax6. GFP expression was sufficiently strong that expressing cells could be distinguished from non-expressing cells using flow cytometry.

**Conclusion:**

This work demonstrates the *functional *conservation of the regulatory elements controlling *Pax6/PAX6 *expression in mice and humans. The transgene provides an excellent tool for studying the functions of different *Pax6/PAX6 *regulatory elements in controlling Pax6 expression in animals that are otherwise normal. It will allow the analysis and isolation of cells in which *Pax6 *is activated, irrespective of the status of the endogenous locus.

## Background

Pax6 is a transcription factor containing an N-terminal DNA binding domain, a paired domain, separated by a glycine-rich linker sequence from a second DNA binding domain, a homeodomain, and a C-terminal proline-serine-threonine-rich transregulatory domain. It is highly conserved in very diverse species. In mammals, it is expressed during development in the eye, in specific regions of the CNS, in the nasal placodes and olfactory epithelium and in the pancreas [1-3]. Haploinsufficiency for *Pax6 *function (*Pax6*^+/-^) in the mouse results in the *Small eye *(*Sey*) phenotype [4]. Homozygotes (*Pax6*^-/-^) die perinatally with no eyes and many brain abnormalities [3-14]. *PAX6 *haploinsufficiency also causes eye and brain defects in humans [15,16].

Normal development requires not only that Pax6 be present in certain cells at certain times but also that it be present in the correct amounts. Schedl et al. [17] showed that severe eye abnormalities are caused not only by under-expression but also by over-expression of Pax6 in mice. Bishop et al. [18,19] and Muzio et al. [20] provided evidence that graded expression of *Pax6 *across the developing neocortex of mice is essential for the correct specification of its major areas. Such findings imply that *Pax6 *expression is tightly regulated and that different levels are maintained in different regions as they grow.

**Figure 1:**
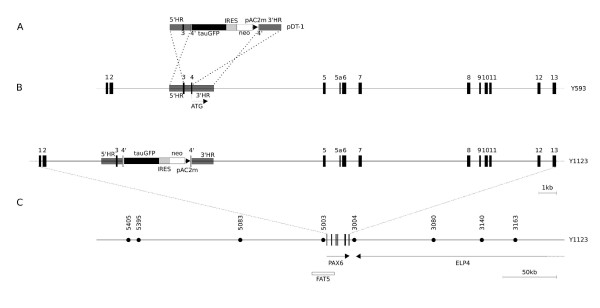
**Generation of YAC Y1123 and DTy54 transgenic mice**. (A) Construct pDT-1 contained sequences expressing tau-GFP and conferring neomycin (neo) resistance, separated by an internal ribosomal entry site (IRES), terminated with polyadenylation and C2MAZ (pAC2m) sequences and flanked by 5' and 3' homology regions (5'HR and 3'HR). (B-C) Homologous recombination resulted in the introduction of this construct at the translation initiation site (ATG) in exon 4 of the human *PAX6 *locus, contained in YAC Y593 [17], to produce YAC Y1123. Successful integration of the modified *PAX6 *locus into the mouse genome was checked on Southern blots using cosmid FAT5 [17] as a probe and by PCR using primers indicated by filled circles in C (sequences in Methods). The modification was designed such that the tau-GFP and neomycin resistance cassettes would be controlled by the gene's regulatory elements and would prevent the production of PAX6 protein from the translational start site in exon 4. The position of the *ELP4 *gene is marked.

Work on humans has indicated that the *PAX6 *regulatory elements extend over more than two hundred kilobases [21-24]. The locus is highly conserved, both in the coding regions (human and mouse proteins are identical) and also in the non-coding regions, where similar long-range control elements have been identified in mouse and *Fugu *by genomic sequence comparisons and DNaseI hypersensitivity analysis [24-26]. A YAC (named Y593) containing 420 kb of the human coding sequence and flanking regions, extending beyond its putative regulatory elements, rescues the mouse *Pax6*^-/- ^phenotype, whereas a YAC containing 110 kb less flanking sequence does not [17,24]. In the work described here, we modified Y593 by introducing tau-GFP and neomycin resistance cassettes so that they would be controlled by the gene's regulatory elements and would prevent the production of PAX6 protein from the translational start site in exon 4 (Fig. 1). This new YAC (called Y1123) was then used to generate transgenic mice.

## Results

### Initial characterization of transgenic mice

Y593 was successfully modified as illustrated in Fig. 1 and as described in Methods to generate Y1123. This YAC was used to generate transgenic mice. Three fertile transgenic founders, named DTy22, DTy42 and DTy54, were identified. They all appeared phenotypically normal and successfully transmitted a tau-GFP-expressing Y1123 transgene to their offspring. PCR with primers marked in Fig. 1 (sequences in Methods) was used to examine the minimum extent of incorporation of Y1123: the results indicated that only DTy54 had incorporated at least the majority of Y1123, whereas DTy22 and DTy42 had incorporated truncated versions. Neither DTy22 nor DTy42 recapitulated the full expression pattern of Pax6. It was possible to identify DTy54 and DTy42 transgenic mice using a hand-held torch emitting blue light, of the wavelength required to excite GFP, and an appropriate filter, as described in [27]. This revealed GFP expression in the eyes of living DTy54 and DTy42 mice; the eyes of DTy22 mice did not express GFP. DTy42 mice expressed GFP only in the eyes. Using DTy54 mice, Y1123 was crossed into embryos that were either *Pax6*^+/- ^or *Pax6*^-/-^. Unlike the unmodified YAC Y593 [17], Y1123 produced no rescue of either the eye or brain defects in these mutants, confirming its predicted lack of function.

Quantitative PCR (qPCR) was used to compare relative fluorescence intensities following amplification with primers specific for human *PAX6 *and mouse *Pax6 *or for human *PAX6 *and mouse *Pax3*. Intensities following amplification for mouse *Pax6 *and *Pax3 *were halved (since there are two copies of each in the mouse genome). The ratios between the intensities from *PAX6 *and half the intensities from *Pax6 *and *Pax3 *are shown in Table 1. We conclude that one copy of Y1123 (or a part of Y1123 in DTy22 and DTy42) had integrated into the genome of each founder. Fluorescent *in situ *hybridization (FISH) using the entire FAT5 cosmid (Fig. 1) as a probe [17] on blood smears from DTy54 showed a single signal per cell, confirming that there were not multiple sites of integration. DTy22 and DTy42 were not studied further here.

**Table 1:** Numbers of copies of integrated transgenes This was estimated from the average of the ratios between fluorescence intensities using primers for human *PAX6 *and 0.5 × the fluorescence intensities using primers for mouse *Pax3 *or *Pax6 *in qPCR reactions on a constant amount of DNA from each of a series of embryos. Each qPCR reaction was repeated three times on each animal.

Lines	Average ratios (± SD)		Numbers of animals
		
	*PAX6*/0.5 × *Pax3*	*PAX6*/0.5 × *Pax6*	
DTy54	0.8 ± 0.2	0.9 ± 0.4	7
DTy22	1.3 ± 0.5	1.0 ± 0.5	6
DTy42	1.4 ± 0.5	1.2 ± 0.5	8

### Expression of tau-GFP in DTy54 mice

**Figure 2:**
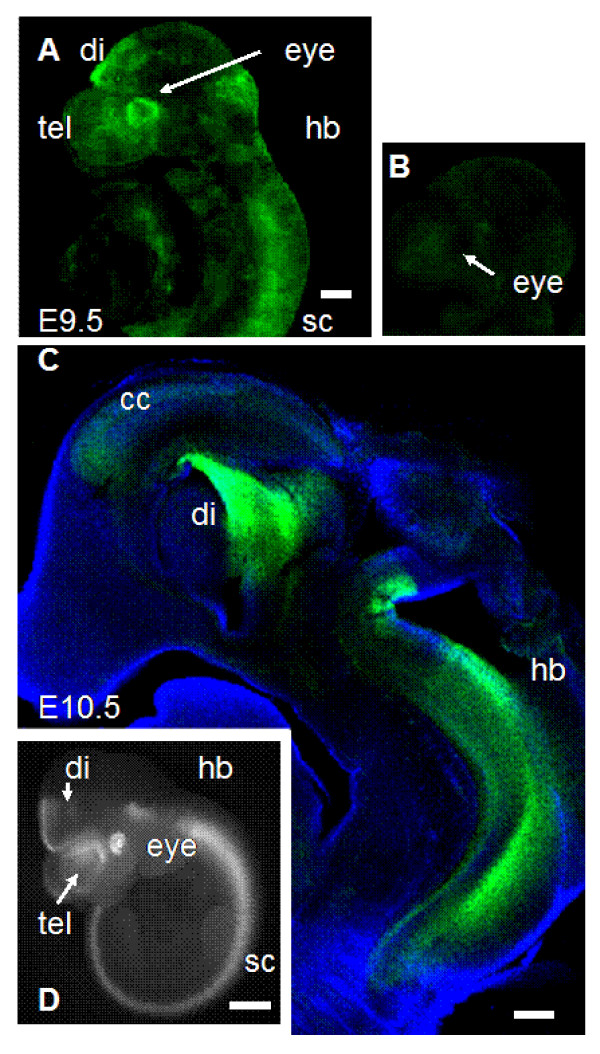
**Expression of tau-GFP in DTy54 transgenic mice at E9.5–10.5**. (A) Whole E9.5 DTy54 embryo showing expression in the eye, forebrain (telencephalon, tel, and diencephalon, di), hindbrain (hb) and spinal cord (sc). (B) Whole E9.5 wild-type embryo showing lack of expression. (C) Parasagittal section through an E10.5 DTy54 embryo showing expression in the cerebral cortex (cc), diencephalon and hindbrain. (D) Whole E10.5 DTy54 embryo showing expression in the eye, forebrain (telencephalon, tel, and diencephalon, di), hindbrain (hb) and spinal cord (sc). Scale bars: A-C, 200 μm; D, 500 μm.

**Figure 3:**
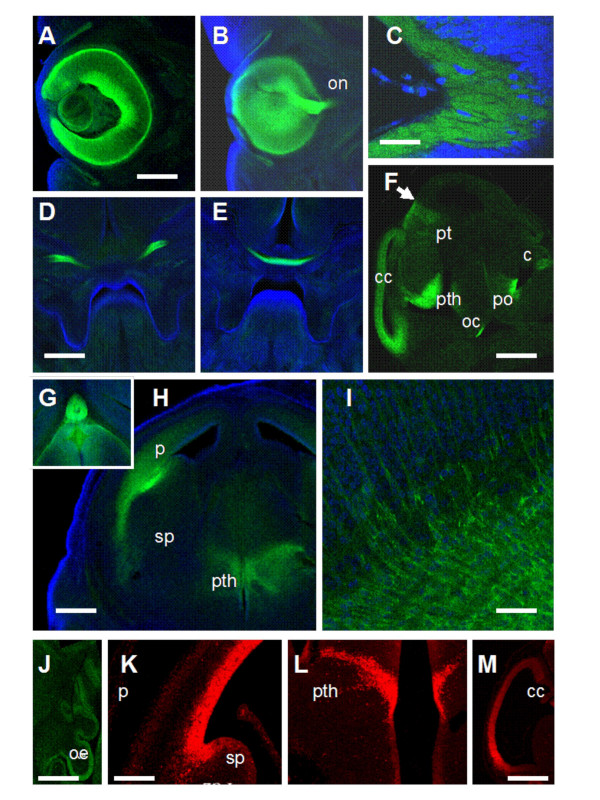
**Expression of tau-GFP in DTy54 transgenic mice at E14.5**. (A-E) Sections showing tau-GFP in the eye and optic tract. The optic nerve (on) contains tau-GFP (B-D). Axons containing tau-GFP are seen emerging from the retina at the optic nerve head (C) and forming the optic chiasm (E). (F) Parasagittal section through the brain at E14.5 showing tau-GFP in the cerebral cortex (cc), prethalamus (pth), pretectum (pt), optic chiasm (oc), basal plate of the pons (po) and primordial cerebellum (c). Arrow points to the posterior boundary of the pretectum. (G-I) Coronal sections at E14.5 showing tau-GFP in the brain: (G) the pineal gland, (H) the pallium (p), subpallium (sp) and prethalamus and (I) the cerebral cortex. (J) Section through the olfactory epithelium (oe). (K-M) Expression of Pax6 shown with immunohistochemistry on coronal sections. Scale bars: A,B 200 μm; C, 20 μm; D,E,G,H,J-L, 300 μm; F,M, 900 μm; I, 50 μm.

To test whether the tau-GFP expression in DTy54 mice was consistent with the established Pax6 expression pattern, embryos were collected at embryonic day (E) 9.5, 10.5, 12.5, 14.5 and 16.5 and eyes were taken from adults. Expression of tau-GFP was seen in the eyes at all ages; examples are shown at E9.5 (Fig. 2A,B), E10.5 (Fig. 2D) and E14.5 (Fig. 3A,B). Expression was present in the retina and lens, as expected, and allowed cellular processes to be visualised due to cytoplasmic labelling by tau-GFP. Particularly striking was label in the axonal projections of retinal ganglion cells, which could be seen in the optic nerve (Fig. 3B–D) and followed through the optic chiasm into the optic tract (Fig. 3E). Elsewhere, tau-GFP expression was confined to regions known to express Pax6 [1,6,7,9,10]. As expected, expression at E9.5–10.5 was in the forebrain (in the telencephalic and diencephalic vesicles), with a sharp posterior boundary of expression at the diencephalic/mesencephalic boundary [7,10], and also in the hindbrain and spinal cord (Fig. 2). Figure 3F shows a parasagittal section through the brain at E14.5: expression was in the cerebral cortex, prethalamus (also known as the ventral thalamus), pretectum, the basal plate in the region of the pons [28] and in the cerebellar primordium [11]. The intensity of label in the cerebral cortex was graded from high rostrally to low caudally (Fig. 3F), in line with the known gradient of expression of Pax6, which is shown using an anti-Pax6 antibody in Fig. 3M[18,20]. Similar to earlier ages, there was a sharp posterior boundary of expression at the border between pretectum and midbrain (arrow in Fig. 3F) [7,10]. In coronal sections, expression of tau-GFP was seen in the pineal gland (Fig. 3G) and nearby in the posterior commissure (not shown), which are also sites of Pax6 expression [14]. There was expression in the prethalamus (Fig. 3H) in a pattern similar to that shown with an anti-Pax6 antibody (Fig. 3L). In the pallium (Fig. 3H), there was a boundary of expression at the border between the pallium and subpallium, in agreement with the known boundary of Pax6 expression (Fig. 3K) [7,10]. Label was seen running ventrally to the amygdaloid region (Fig. 3H), mirroring the known expression of Pax6 in this area [9,29]. In the cerebral cortex, tau-GFP was seen in the radial processes of cells located on the ventricular side of the cortical wall (Fig. 3I); again, this was anticipated since Pax6 is known to be expressed in radial glial cells [30]. There was expression in the olfactory epithelium (Fig. 3J) [1,2].

**Figure 4:**
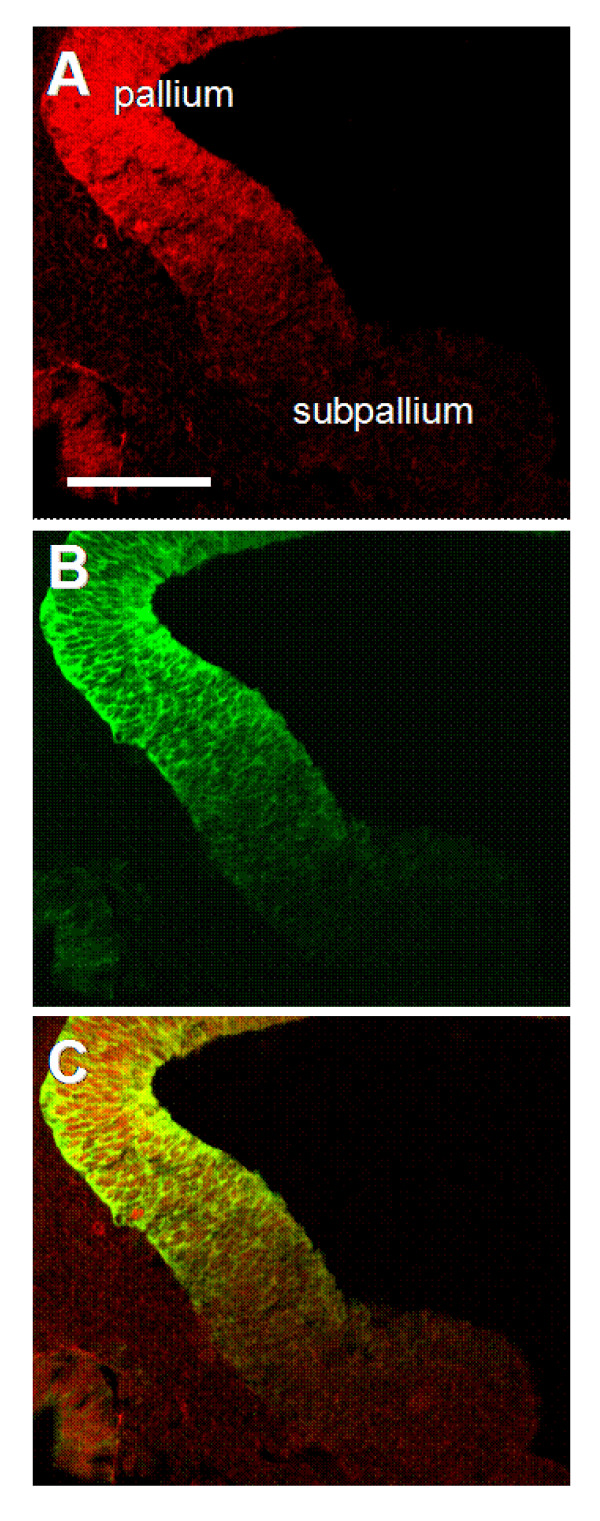
**Expression of tau-GFP in DTy54 transgenic mice at E12.5**. Coronal sections through the pallial/subpallial border of E12.5 embryos stained with antibodies against Pax6 (A) and GFP (B); images are merged in C. Scale bar: 200 μm.

Immunhistochemistry with anti-Pax6 and anti-GFP antibodies confirmed the presence of GFP in Pax6-expressing regions. An example of co-localization at E12.5 is shown in Fig. 4: Pax6 is expressed on the pallial side of the pallial/subpallial border (Fig. 4A), as is tau-GFP (Fig. 4B,C). Overall, we concluded that the patterns of label with tau-GFP are exactly as anticipated on the basis of the known expression of Pax6 and that regional differences in the intensity of label in the cerebral cortex reflect known differences in the level of expression of Pax6.

### Analysis of DTy54 brains with flow cytometry

**Figure 5:**
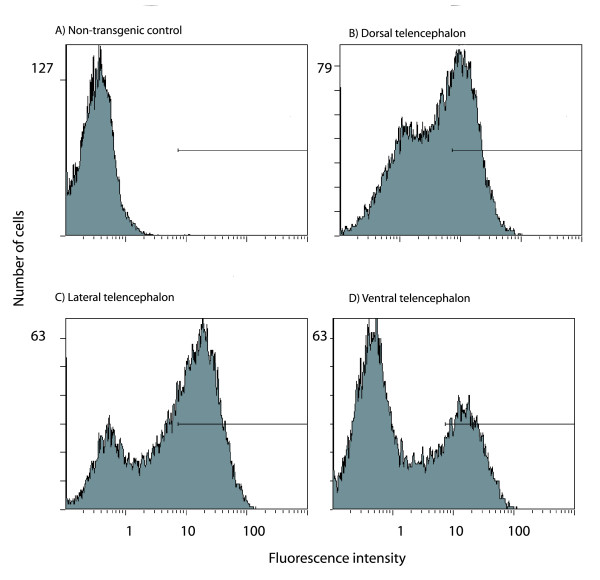
**Expression of tau-GFP in DTy54 transgenic mice at E14.5 quantified with flow cytometry**. Frequency histograms of cell number against GFP fluorescence for samples of cells from (A) the telencephalon of non-transgenic controls and (B-D) the dorsal, lateral and ventral parts of DTy54 embryos.

One of the potential uses of this transgenic mouse is to allow the isolation of Pax6-expressing cells. We demonstrated that this is possible using flow cytometry on dissociated cells from the brains of E14.5 embryos. The telencephalic vesicles were removed and each was cut into dorsal, lateral and ventral components. Data are shown in Fig. 5. Analysis of non-transgenic embryos provided frequency distributions of background fluorescence intensity (Fig. 5A). A gate was set to cover intensities above the upper limit of the fluorescence seen in these controls, on the basis that cells falling within this gate in transgenic embryos (Fig. 5B–D) were certain to be expressing tau-GFP. In samples from dorsal telencephalon of DTy54 embryos (Fig. 5B), a large proportion of cells had fluorescence levels within the gate. There were also large numbers of cells whose fluorescence intensities were not within the gate but were higher than the average intensity in non-transgenic controls. It is likely that these are cells expressing GFP at lower levels; for example, many may be in the process of down-regulating the transgene as they differentiate, which is the pattern of expression of Pax6 [1,6,12]. A similar picture was seen in samples from the lateral and ventral telencephalon (Fig. 5C,D). The average fluorescence intensity of cells within the gate was higher in the lateral telencephalon than in the dorsal telencephalon (peak shifted to the right in Fig. 5C compared to Fig. 5B), which agrees with the brighter fluorescence of cells in this region in sections (Fig. 3H). The proportion of cells within the gate was smallest in samples from the ventral telencephalon (Fig. 5D), which agrees with the fact that fewer cells in this region express Pax6 (Fig. 3H).

## Discussion

In DTy54, the modified YAC that had integrated into the mouse genome did not affect the endogenous *Pax6 *locus, unlike an alternative strategy involving the insertion of a reporter gene into the endogenous locus [3]. The YAC1123 transgene can be crossed onto mice with any *Pax6 *status (e.g. *Pax6*^+/+^, *Pax6*^+/-^, *Pax6*^-/-^, *Pax6*^*loxP*/*loxP*^) to identify and isolate those cells in which *Pax6 *is being activated by upstream factors. In addition to generating a useful new tool for understanding the role of *Pax6*, our results demonstrate that the elements regulating the human *PAX6 *gene present in Y1123 and Y593 [17] are necessary and sufficient to recapitulate accurately the expression of *Pax6 *in mice. This indicates that these elements are not only structurally [24,25] but also functionally highly conserved. In their original study of mice containing human *PAX6*-expressing YACs, Schedl et al. [17] suggested functional conservation of the regulatory elements controlling the human and mouse genes on the basis that the human locus is able to complement the *Sey *mutation in mouse. The introduction of PAX6-producing transgenes corrected the eye defects in heterozygotes and rescued homozygotes from perinatal death. It remained unclear, however, how accurately the human regulatory elements reproduce the pattern of endogenous mouse *Pax6 *expression. Although Y593 must have caused re-expression of the missing factor in those cells that normally express it, thereby rescuing their abnormal phenotypes, additional ectopic expression from Y593 might have gone undetected. Our current work complements that of Schedl et al. [17] by demonstrating a remarkable conservation of function of the *Pax6*/*PAX6 *regulatory elements in the two species.

**Figure 6:**
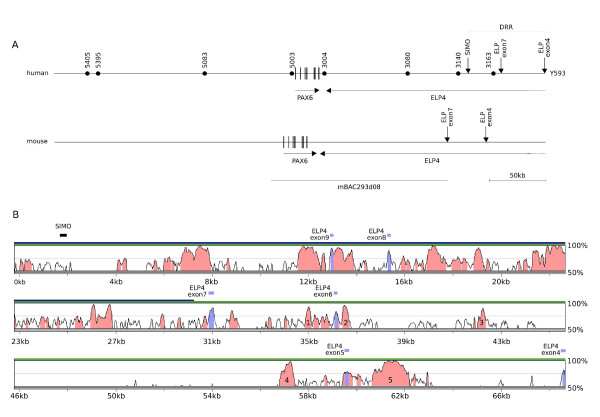
**Genomic comparison of YAC Y593 and BAC mBAC293d08**. (A) The *PAX6 *gene is located centrally in Y593 [17]. The 3' end of Y593 is marked by exon 4 of the *ELP4 *gene. YAC Y593 has not been end-sequenced but is known to reside between the STS markers AFM324yh5 ~20 kb 5' and D11S4662 ~17 kb 3' to the YAC and is approximately 420 kb in length. The DRR (downstream regulatory region) is defined at the 5' end by the SIMO breakpoint and at the 3' end by *ELP4 *exon 4 [31]. Mouse BAC mBAC293d08 spans the region 12 kb 5' to exon 0 of *Pax6 *through to approximately 0.6 kb before *ELP4 *exon7 and is 160 kb in length [31]. It therefore lacks the genomic region between *ELP4 *exons 4 and 7 that comprise part of the DRR region in the human. (B) LAGAN/VISTA pairwise alignment of human and mouse genomic intervals spanning the region from SIMO to *ELP4 *exon 4 [41,42]. The 3' extent of mBAC293d08 is shown by the blue bar and of Y593 by the green bar. Regions shaded pink demark regions of >50% sequence identity over a window of 100 bp. In the 3' region common to Y593 and Y1123, but absent from mBAC293d08, rankVISTA analysis identifies five non-coding regions that are evolving more slowly than a modelled neutrally evolving base sequence (1: 199 bp p = 2.2e-03, 2: 466 bp p = 1.3e-07, 3: 223 bp p = 8.3e-04, 4: 509 bp p = 1.0e-12 and 5: 1564 bp p = 1.6e-42) [43]. These highly conserved regions could contain regulatory elements responsible for differences in expression between the transgenic reporter mice carrying mBAC293d08 and Y1123.

Recently, Kim and Lauderdale [31] described the generation of a bacterial artificial chromosome (BAC) transgenic reporter mouse containing 160 kb of mouse genomic DNA from around the mouse *Pax6 *gene. Unlike the YAC transgene described here, the BAC transgene did not generate expression in diencephalic and olfactory cells that are known to express Pax6. A likely explanation for this difference is that the shorter BAC transgene is missing some important regulatory elements. Figure 6 compares YAC Y593 (which spans the same genomic interval as its derivative, YAC Y1123) with BAC mBAC293d08 [31]. BAC mBAC293d08 lacks the genomic region between *ELP4 *exons 4 and 7 that comprises part of the downstream regulatory region (DRR) in the human. LAGAN/VISTA pairwise alignment identifies five highly conserved regions that could contain regulatory elements responsible for differences in expression between the transgenic reporter mice carrying mBAC293d08 and Y1123 (Fig. 6).

## Conclusion

This work provides further evidence that the *Pax6*/*PAX6 *regulatory elements are highly conserved not only structurally but also functionally in mice and humans. Y1123 provides an excellent tool for studying the functions of different *Pax6/PAX6 *regulatory elements and will allow the analysis and isolation of cells in which *Pax6 *is activated, irrespective of the status of the endogenous locus.

## Methods

### Generation of the DTy54 transgenic mouse

All work on mice followed current Home Office (UK) regulations stipulated in the Animals (Scientific Procedures) Act 1986. An overview of the strategy is illustrated in Fig. 1. We inserted a tau-GFP reporter cassette and a neomycin resistance cassette, linked by an internal ribosomal entry site (IRES), in frame into the translation start site in exon 4 of the *PAX6 *gene in YAC Y593 [17] by homologous recombination using a yeast URA3 selectable marker. The manipulated YAC (named Y1123) was then used to generate transgenic mice.

#### Integration of YAC Y593 into yeast window strain W3

Before modifying the parental YAC Y593 with the reporter construct, it was introduced into a yeast window strain. This was necessary because Y593 co-migrates with similar sized endogenous yeast chromosomes in pulse field gel electrophoresis, making it difficult to isolate from the endogenous chromosomes. Each window strain contains defined alterations in its karyotype, which provide a large size interval, or window, devoid of endogenous chromosomes [32,33]. Window strain W3 was mated with Y593 using the *kar*-cross method [34,35]. Y593, in addition to the *PAX6 *gene locus, contains the genes allowing yeast cells to produce adenine and tryptophan. By removing adenine hemisulfate salt and tryptophan from the growth medium, it was possible to select for yeast colonies expressing these genes and, therefore, containing Y593.

#### Integration of reporter cassette into Y593 by homologous recombination

**Figure 7:**
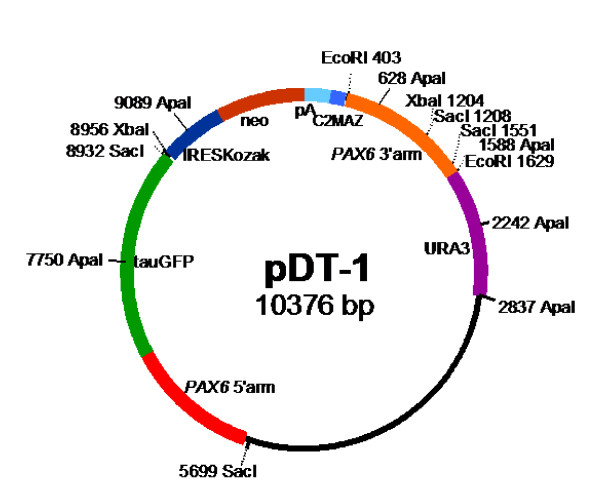
**Map of plasmid pDT-1**. The components of the construct shown in Fig. 1A and the URA3 sequence are marked.

Y593 was modified to generate a new YAC (named Y1123) using the plasmid pDT-1 (Fig. 7), which contained the following elements (Figs. 1, 7). (i) Coding sequence for tau-GFP fusion protein; the microtubule binding protein tau would allow the visualisation of the processes of expressing cells [13]. (ii) An IRES followed by an optimised Kozak translation consensus start site (IRESKozak) to allow the translation of two cis genes from a single transcript [36]. (iii) A neomycin resistance (neo) cassette to allow G418-based selection of expressing cells. (iv) A polyadenylation (pA) site to allow polyadenylation of the tau-GFP-IRES-neo mRNA. (v) A C2MAZ site to slow RNA polymerase II [37] and promote transcription termination; the aim was to further reduce the chances of transcription of the entire targeted locus, which might have reduced marker expression through splicing around exon 4.

Once Y593 had been moved into the window strain it was transformed with the bacterial construct pDT-1 using modified lithium acetate yeast transformation. W3 has a defective URA3 gene and is unable to survive pyrimidine starvation. Since pDT-1 contained the yeast gene URA3 (Fig. 7), any cell harbouring Y593 into which pDT-1 had recombined survived pyrimidine starvation and was selected by the omission of uracil from the growth medium.

Several colonies were picked and screened for the likely presence of a complete pDT-1 using PCR for parts of pDT-1 distant from the URA3 gene and with Southern blots. Of 18 clones screened, one showed correct first round recombination. This clone was grown in the presence of 5-fluoroorotic acid (5-FOA), which prevents yeast cells containing the URA3 gene from growing and provides selection for removal of the URA3 gene by internal homologous recombination [38,39]. Nine clones were picked from the 5-FOA plate, designated 1121 to 1129, and Southern blots were done to identify correct clones. One clone (1123) was identified as correct, giving a success rate of about 11%. PCR combined with restriction digests on some of the PCR products was used to confirm that the individual parts of the reporter cassette were present in the clone. The junction between *PAX6 *and tauGFP was checked by sequencing in both directions, confirming that the *PAX6 *ATG in exon 4 was followed immediately by tauGFP.

#### Microinjection of Y1123 and initial assessment of transgenic mice

Y1123 DNA was isolated for microinjection using alternating contour-clamped homogeneous electric field pulse field gel electrophoresis [40]. Injected one-cell embryos (from crosses of C57Bl/6 and CBA mice) were either replaced immediately into pseudopregnant female mice or first cultured overnight until two-cell. About 5% of injected one-cell embryos were born. Subsequent breeding was such that all mice carrying Y1123 studied here were hemizygous for the transgene. Southern blotting with a full-length cDNA probe was used to confirm that the modified *PAX6 *coding region had integrated. PCR with primers shown in Fig. 1 was used to confirm the extent of incorporation of Y1123. The primer sequences were:

3163F AAGCCATTTTGTTGGTGAGC

3163R TTCCAGTTATACAGGGGCTGA

3140F AAGGTGCCCAGCCTAATTCT

3140R TCGTCTCGATCTCCTGACCT

5003F CAGAGGGAGGACCTCTCAGG

5003R TTTGCCTTTAGGGCTCACTG

3004F CTTCCCTGGCTACCATGTCT

3004R CGGCCCAGTGAATTAGAAAA

3080F TGAAAATGCAAACAGGTTCC

3080R AAGCCGTCAGACCACTTTTG

5083F TGAGAGCTGTGCAGAGCAGA

5083R GAAAGCAAAACCCTGGACAA

5405F GCCATCTGAAAGCTGAGGAG

5405R CCAGCCTACCTTGACATGCT

5395F GACACGCTGGTCACCAAGTA

5395R TTACAGCGGACCCCTCTTC

FISH on blood smears, with cosmid FAT5 as a probe (marked as CFAT5 in Fig. 1) and methods described in Schedl et al. [17], was used to search for possible multiple integration sites. Quantitative PCR (qPCR; QuantiTect SYBR Green PCR Kit, Qiagen) was used to identify the number of copies of Y1123 present in the genome, using three sets of primers, one specific for human *PAX6*, one specific for mouse *Pax6 *and one specific for mouse *Pax3*. The latter two sets were standards (detecting genes with copy numbers of 2) against which to compare the intensity of the product from Y1123. The three sets of primers were as follows: (i) human *PAX6 *specific primers (*PAX6*HumF CCGTGTGCCTCAACCGTA, *PAX6*HumR CACGGTTTACTGGGTCTGG); (ii) mouse *Pax6 *specific primers (*Pax6*MouF CGCAAATACACCTTTGCTCA, *Pax6*MouR GAGGGTTTCCTGGATCTGG); (iii) mouse *Pax3 *specific primers (*Pax3*MouF AAGCAGCGCAGGAGCAGAACC, *Pax3*MouR CCTCGGTAAGCTTCGCCCTCT). These three sets of primers allowed the amplification of sequences of similar length with similar reaction kinetics. Conditions were such that the fluorescence intensity was related linearly to the amount of starting DNA; once this had been established, 300 ng of DNA was used in each qPCR reaction. By comparing the fluorescence generated with sets two and three against the fluorescence generated when the same amount of DNA was used with set one, the number of *PAX6 *gene copies was calculated. Each reaction was repeated three times on each of a series of embryos.

### Assessing the transgene's expression

Pregnant females were killed at various ages by cervical dislocation and embryos were fixed overnight in ice cold 4% paraformaldehyde and embedded in 4% low melting point agarose (the day of the vaginal plug was designated E0.5). Vibratome sections were cut at 200 μm, counterstained with TOPRO3 (Molecular Probes, NL), mounted on glass slides and imaged using a Leica confocal microscope. For immunohistochemistry, tissue was fixed overnight in 4% paraformaldehyde, transferred to 15% sucrose, embedded in 7.5% gelatin/15% sucrose in phosphate buffered saline and placed in 30% sucrose overnight. Cryostat sections (15 μm) were cut and transferred to 20% goat serum in phosphate buffered saline containing 0.1%Triton-X for 30 min at room temperature. Sections were incubated with mouse anti-Pax6 ascites (1:5000; Developmental Studies Hybridoma Bank) and rabbit anti-GFP antibody (1:10000; Abcam) overnight at 4°C and for 1 hr at room temperature the following day. Secondary antibodies were goat anti-mouse and goat anti-rabbit Alexa fluor 568 and 488 respectively (1:150; Molecular Probes), applied for 1 hr at room temperature.

### Flow cytometry

Telencephalic tissue from E14.5 wild-type and DTy54 embryos were dissociated with papain (Papain Dissociation System, Worthington Biochemical). Cells in suspension were analysed on a Beckman-Coulter XL flow cytometer (10,000–20,000 cells were analysed per sample).

## Abbreviations

BAC, bacterial artificial chromosome; c, cerebellum; cc, cerebral cortex; di, diencephalon; DRR, downstream regulatory region; E, embryonic day; 5-FOA, 5-fluoroorotic acid; FISH, fluorescent in situ hybridization; GFP, green fluorescent protein; hb, hindbrain; HR, homology region; IRES, internal ribosomal entry site; neo, neomycin resistance; oc, optic chiasm; oe, olfactory epithelium; on, optic nerve; p, pallium; pA, polyadenylation; pt, pretectum; pth, prethalamus; po, pons; sc, spinal cord; sp, subpallium; tel, telencephalon; YAC, yeast artificial chromosome.

## Authors' contributions

D. Tyas did most of the work towards generating the transgenic mouse, T.I. Simpson helped design and supervise the work, C.B. Carr did some of the expression analysis, D.A. Kleinjan collaborated on the generation of the mouse and V. van Heyningen, J.O. Mason and D.J. Price participated in design and supervision and in writing the manuscript.
